# Mineral substrate quality determines the initial soil microbial development in front of the Nordenskiöldbreen, Svalbard

**DOI:** 10.1093/femsec/fiad104

**Published:** 2023-09-02

**Authors:** Petra Luláková, Hana Šantrůčková, Josef Elster, Martin Hanáček, Petr Kotas, Travis Meador, Václav Tejnecký, Jiří Bárta

**Affiliations:** Department of Ecosystem Biology, Faculty of Science, University of South Bohemia, Branišovská 31a, 37005 České Budějovice, Czech Republic; Department of Ecosystem Biology, Faculty of Science, University of South Bohemia, Branišovská 31a, 37005 České Budějovice, Czech Republic; Institute of Botany ASCR, Dukelská 135, Třeboň, Czech Republic; Centre for Polar Ecology, Faculty of Science, University of South Bohemia, Na Zlaté Stoce 3, 37005 České Budějovice, Czech Republic; Polar-Geo-Lab, Department of Geography, Faculty of Science, Masaryk University, Kotlářská 267/2, 611 37 Brno, Czech Republic; Centre for Polar Ecology, Faculty of Science, University of South Bohemia, Na Zlaté Stoce 3, 37005 České Budějovice, Czech Republic; Department of Ecosystem Biology, Faculty of Science, University of South Bohemia, Branišovská 31a, 37005 České Budějovice, Czech Republic; Department of Ecosystem Biology, Faculty of Science, University of South Bohemia, Branišovská 31a, 37005 České Budějovice, Czech Republic; Institute of Soil Biology and Biogeochemistry, Biology Centre Czech Academy of Sciences, Na Sádkách 702/2, 37005 České Budějovice, Czech Republic; Department of Soil Science and Soil Protection, Faculty of Agrobiology, Food and Natural Resources, Czech University of Life Sciences in Prague, Kamýcká 129, Prague, Czech Republic; Department of Ecosystem Biology, Faculty of Science, University of South Bohemia, Branišovská 31a, 37005 České Budějovice, Czech Republic; Centre for Polar Ecology, Faculty of Science, University of South Bohemia, Na Zlaté Stoce 3, 37005 České Budějovice, Czech Republic

**Keywords:** Arctic soils, biogeochemistry, deglaciation, glacier forefield, microbial assembly, soil succession

## Abstract

Substrate geochemistry is an important factor influencing early microbial development after glacial retreat on nutrient-poor geological substrates in the High Arctic. It is often difficult to separate substrate influence from climate because study locations are distant. Our study in the retreating Nordenskiöldbreen (Svalbard) is one of the few to investigate biogeochemical and microbial succession in two adjacent forefields, which share the same climatic conditions but differ in their underlying geology. The northern silicate forefield evolved in a classical chronosequence, where most geochemical and microbial parameters increased gradually with time. In contrast, the southern carbonate forefield exhibited high levels of nutrients and microbial biomass at the youngest sites, followed by a significant decline and then a gradual increase, which caused a rearrangement in the species and functional composition of the bacterial and fungal communities. This shuffling in the early stages of succession suggests that high nutrient availability in the bedrock could have accelerated early soil succession after deglaciation and thereby promoted more rapid stabilization of the soil and production of higher quality organic matter. Most chemical parameters and bacterial taxa converged with time, while fungi showed no clear pattern.

## Teaser

Different substrate geochemistry on two adjacent glacier forefields drives early soil succession, providing limiting nutrients for microorganism assembly.

## Introduction

Retreating ice fronts, induced by global climate change, have increasingly exposed landscapes in polar and alpine regions, leading to microbial and plant colonization and soil formation (Hodkinson et al. 2003, Bajerski and Wagner 2013, Bradley et al. 2014). Microbes are the pioneer colonizers of these deglaciated barren surfaces (Schütte et al. 2010, Bradley et al. 2014). Their distributions are determined by nutrient availability, and at the same time, they drive nutrient and mineral cycling during the initial phases of soil stabilization and plant establishment (Hodkinson et al. 2003, Tscherko et al. 2003, Borin et al. 2010). As such, chronosequence approaches have provided a useful tool to investigate the environmental factors that may affect the microbial community during ecosystem development (Bernasconi et al. 2011, Zumsteg et al. 2012, Knelman et al. 2014, Garrido-Benavent et al. 2020).

Plant colonization and plant–microbe interactions have been identified as the predominant factors that shape microbial community composition and nutrient accumulation along alpine and low latitude chronosequences (Tscherko et al. 2003, Zumsteg et al. 2012, Brown and Jumpponen 2014). In comparison, polar forefields mainly comprise cryptograms, lichens and bryophytes (Garrido-Benavent et al. 2020), and may remain mostly devoid of plants even a century after glacier retreat (Bradley et al. 2016). Even without vascular plants, shifts of microbial community occur in polar regions over short time intervals (Nemergut et al. 2007, Garrido-Benavent et al. 2020, Vimercati et al. 2022), though the possible abiotic driving factors and their complex interaction with carbon sources are still being debated (Tscherko et al. 2003, Knelman et al. 2014). Together with plants, soil age has also been suggested to be a key factor affecting the microbial life and edaphic properties (Noll and Wellinger 2008, Bradley et al. 2014), though environmental disturbances may overprint the effect of time (Kim et al. 2017). The selective pressure of the environmental factors (local geographic and climatic conditions) and the physico-chemical properties of the substrate (e.g. soil texture and porosity, nutrient status, pH, and mineralogical properties) (Lazzaro et al. 2010) together with accessibility to pioneer sites (Jumpponen et al. 1999) affect substrate availability and thereby microbial distribution in the glacier forefield (Zhou et al. 2002). In general, the initial phases of succession select for microbes that are able to fix carbon and nitrogen or otherwise gain the nutrients from weathering (Wojcik et al. 2019), together with heterotrophs thriving on available organic nutrient sources (Bardgett et al. 2007, Schulz et al. 2013). The proportion and decomposition of old, microbial necromass gradually increases, leading to the recycling of accumulated nutrients (Bradley et al. 2014). Recently, among all the possible drivers, low nutrient availability was suggested to be the main constraining factor for microbial community development, which may rival other edaphic properties and harsh climatic conditions of glacier forefields (Knelman et al. 2014, Schmidt et al. 2016, Darcy et al. 2018). That is, a lack of available nutrients could impose stronger constraints on microbial community development in early succession than the absence of carbon substrates (i.e. energy source) (Castle et al. 2017). The main factors that determine nutrient availability of newly exposed oligotrophic polar soils remain largely unexplored but are presumably related to soil bedrock type (Barton et al. 2007, Lazzaro et al. 2009, Tytgat et al. 2016, Wojcik et al. 2019) and/or soil organic matter sources (Fierer et al. 2010).

Bedrock type not only determines physical properties of the landscape, such as texture, porosity (Lazzaro et al. 2009), and the rate of nutrient release due to weathering (Tytgat et al. 2016), but also provides minerals that are known to select for specialized bacteria (Carson et al. 2009, Meola et al. 2014). In this way, the bedrock type controls microbial habitat and release of nutrients, and thereby significantly influences microbial activity in the initial, plant-free phases of soil development (Carson et al. 2009). In particular, phosphorus availability has been suggested to limit succession in cold and arid climates with low weathering rates (Darcy et al. 2018).

The interplay of life strategies employed by microbial colonizers is depicted by the distinct successional trajectories that have been described for bacteria and fungi (Rime et al. 2016b, Jiang et al. 2018, Garrido-Benavent et al. 2020, Vimercati et al. 2022). This may be caused by ecological differences, such as substrate utilization or stress tolerance of fungi and bacteria (Brunner et al. 2011, Hannula et al. 2017), which are reflected in the highly specific requirements (Rime et al. 2016b) and more random distribution (Brown and Jumpponen 2014) of fungi in early succession compared to bacteria. Accordingly, taxonomically diverse bacterial communities evolving on unrelated glacier forefields with different geochemistries have been shown to converge with increasing age of succession (Castle et al. 2016), while fungi do not exhibit consistent patterns (Brown and Jumpponen 2014).

The present study combines fungal and bacterial successional pathways together with substrate geochemistry to improve our understanding of glacial forefield colonization processes and the implications for future glacier retreat. While most geographical and soil forming conditions of the two forefield chronosequences in front of Nordenskiöldbreen (Svalbard, Norway) are similar, differences in mineral substrate composition offer the possibility to test the effects of contrasting bedrock type and glacial landscape. We, therefore, sampled along the contrasting 80-year successional High Arctic glacial forefield gradients, with the assumption that the controls imposed by physico-chemical properties of the mineral substrate on bacterial and fungal successional trajectories are mitigated with soil age.

Our hypotheses are:

- There is a significant influence of mineral substrate and different quality of organic matter on the microbial assembly in the first stages of the soil development.
- The influence of the mineral substrate and organic matter on the microbial assembly is mitigated with soil age and the patterns of response are different for bacteria and fungi.

## Materials and methods

### Study sites and sample collection

Nordenskiöldbreen (central Spitsbergen, 78.67° N, 16.78° E) is a polythermal valley glacier and largest outlet glacier of Lomonosovfonna Ice Cap in Billefjorden area (Ewertowski et al. 2016, Allaart et al. 2018). The middle part of glacier tongue is a tidewater terminus and both lateral margins are terrestrial. Nordenskiöldbreen has been retreating since the Little Ice Age (LIA) maximum at the end of the 19th century (Allaart et al. 2018). Between 1896 and 2013, the northern terrestrial terminated margin retreated about 1.4 km, while the southern terrestrial terminated margin retreated about 3.5 km (Ewertowski et al. 2016). The newly exposed glacier forefields rises from 2 to 35 m a.s.l. The southern and northern forefields differ in bedrock geology and glacial landscape. The northern *silicate* forefield is made up of metamorphic rocks (primarily mica schist, calc-pelitic schists, and marble). The relief is dominated by ice-moulded bedrock, which is covered by a thin and discontinuous glacigenic and glaciofluvial deposits comprising monotonous clastic material (mainly gneiss and other metamorphites) (Dallmann et al. 2004, Allaart et al. 2018). The southern *carbonate–silicate* forefield is flat and made up of sedimentary carbonates and fewer mica schists. The bedrock is almost completely covered by thick glacigenic deposits of variable and exotic clastic compositions (granitoids, mafic magmatite, and diverse metamorphites; Dallmann et al. 2004).

The annual mean surface ground temperature of the Billefjorden tundra, referred to as a reference site, is −5.2°C, ranging between −19.5°C for April and 10.8°C for July, and the annual precipitation is typically less than 200 mm (Førland et al. 2011, Láska et al. 2012). Vegetation cover at both forefields is initially absent or sparse (0%–20% vegetation cover, data not shown), consisting primarily of soil crusts and/or single pioneer plants (e.g. *Saxifraga oppositifolia, Braya purpurascens, Salix polaris*, and *Dryas octopetala)* on wetter and wind shielded spots. Tundra reference sites are covered predominantly by *Carex* spp. grasses and woody shrubs (*S. polaris* and *D. octopetala)*.

**Figure 1.**
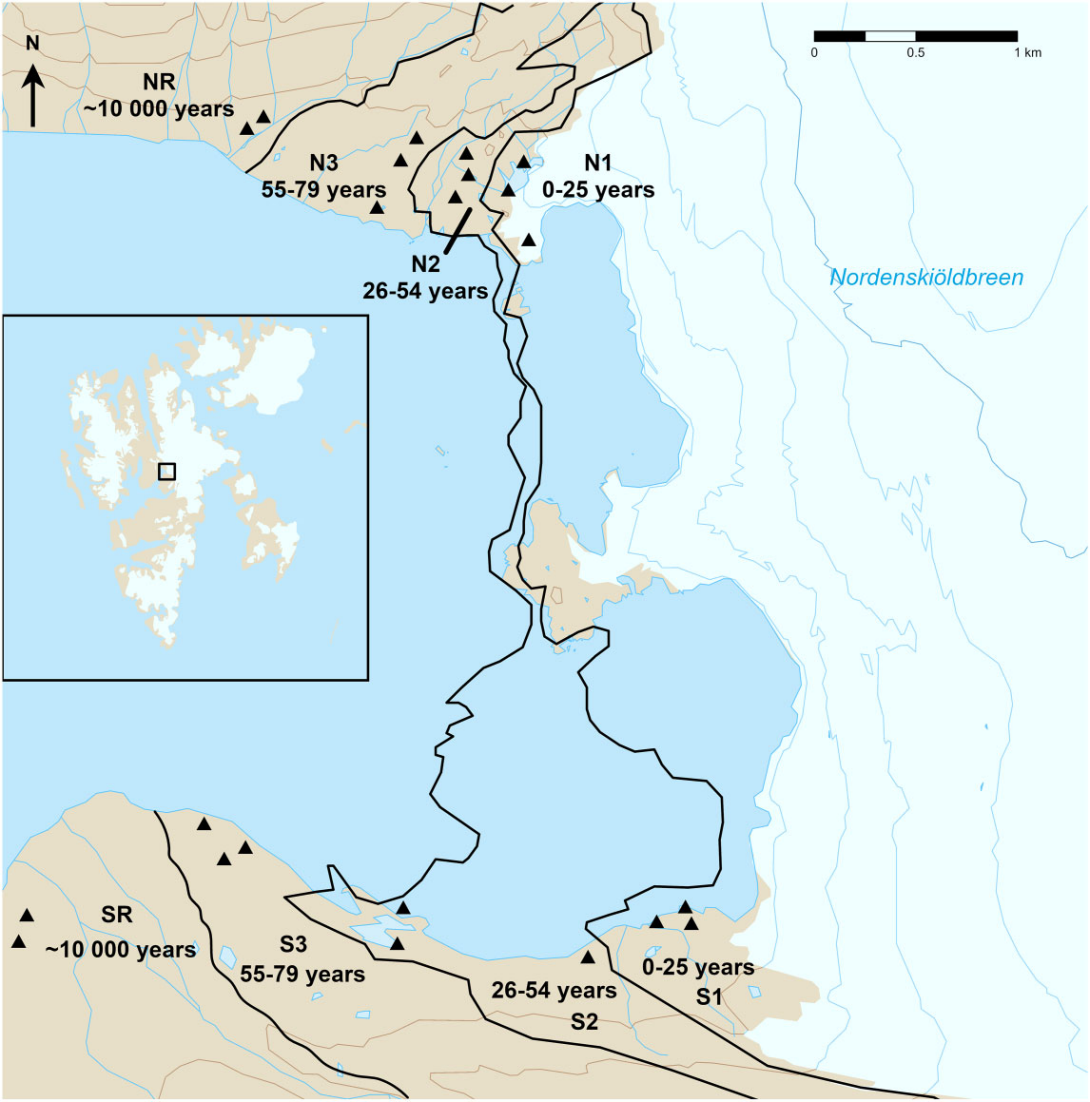
Northern and southern forefield of the Nordenskiöldbreen with black triangles as sampling sites and marked zones of deglaciation based on the satellite imagery provided by © Norsk Polar Institute (http://www.npolar.no) and map background from TopoSvalbard (http://toposvalbard.npolar.no). The Nordenskiöldbreen glacier front is from summer 2009 and N1 sites were already deglaciated during the sampling campaign.

Soils were collected in July 2015 and 2016 from both northern *silicate* (N1, N2, N3, and NR sites) and southern *carbonate–silicate* (S1, S2, S3, and SR sites) forefields with a chronosequence approach (Crocker and Major 1955, Nemergut et al. 2007, Bernasconi et al. 2011). A total of four zones with increasing age were sampled from the glacier front (I. 0–25, II. 26–54, and III. 55–79 years old; Fig. 1) to the tundra ‘reference site’ behind the glacier front moraine from the end of the LIA (IV. 10 000 years). Zones were determined in ArcMap 10.6.1 (ESRI 2018) based on aerial photographs from the Norwegian Polar Institute from the years 1936, 1961, 1990, and 2009 (Norsk Polar Institute 2018). Proglacial systems are characterized by high spatial variability and redeposition (Hodkinson et al. 2009, Bernasconi et al. 2011), therefore, the sampling sites were chosen carefully with an effort to find stable surfaces. The sites were first randomly chosen in ArcMap, checked for potential disturbances by glacier streams on the old aerial photos and then again examined by a geologist in the field with the special focus on the visible glacier movement. Mineral surface soil samples (top 5 cm) were collected from unvegetated areas or after the removalof soil crusts when present. At every site, three independent soil samples were pooled from four subsamples, each one meter apart (all together 66 mixed samples, 33 for each forefield; Fig. 1). To capture approximate volumetric soil moisture, we also collected 10 cm × 10 cm × 5 cm cubes into plastic bags.

Bulk soil was homogenized and sieved with a 2-mm sieve and immediately analyzed for available forms of nitrogen (N-NO_3_^−^, N-NO_2_^−^, and N-NH_4_^+^—the sum is further presented as mineral nitrogen, Nmin) and for available phosphorus (P-PO_4_^3−^). Water soil extracts in the ratio 1:5 were prepared to analyze DOC and kept frozen until measured. Part of the soil was immediately frozen for DNA analyses, enzyme activities, and biomarker assessment, and part was air-dried for further physical and chemical analyses. The rest was kept at 4°C until processing for microbial biomass within 1 month. The bulk density samples were dried for 24 h at 105°C in the oven, sieved for 2 mm fraction, and weighted immediately after sampling and drying.

### Soil biogeochemistry and microbial quantity

Dissolved organic carbon (DOC) was extracted with cold water (sample: water ratio of 1:5) by shaking at 20°C for 1 h and subsequently analyzed after transport for total organic carbon on a LiquiTOC II (Elementar, Germany). Water extractable forms of inorganic nitrogen (N-NO_2_^−^, N-NO_3_^−^, and N-NH_4_^+^; sample: water ratio 1:5) were determined immediately after sampling on the field station using photometric nitrite, nitrate, and ammonium test kits (Spectroquant Test, Merck, Darmstadt, Germany) and a field portable spectrophotometer (Hach Lange DR 2800, Germany). Their concentrations were summed and referred to as mineral nitrogen (Nmin). Water extractable phosphorus (P-PO_4_^3−^) was determined as soluble reactive phosphorus according to Murphy and Riley (1962) using a field portable spectrophotometer (Hach Lange DR 2800).

Total content of elements in soil matrix (Si, Ca, Al, K, Fe, Mg, P, and S) was determined by X-ray fluorescence (XRF) (Delta Premium XPD 6000, Olympus Innov-X, USA) under standard lab conditions (Tejnecký et al. 2015). Labile compounds (Ca^2+^, K^+^, Mg^2+^, and SO_4_^2−^) were extracted by cold water (sample: water ratio 1:10) and pH of solution (active pH) was measured potentiometrically (pH meter inoLab pH Level 1 WTW, Germany). The concentration of extracted cations was determined by means of ICP-OES (iCAP 7000, Thermo Scientific, USA) and content of SO_4_^2−^ was determined by means of ion chromatography (ICS 1600, Dionex, USA).

The amount of 10 g of the 2-mm sieved sample was air-dried and treated with 30% hydrogen peroxide and the soil suspension was allowed to react under heated conditions with subsequent additions of 30% hydrogen peroxide, until all soil organic matter was removed. The soil suspension was then measured by laser diffraction method (LDM) using a Fritsch Analysette 22 MicroTrec plus in the range of 0.1–2000 µm (ISO 11277 2009). As the amount of clay particles was overestimated by the Fraunhofer theory, the pycnometer bottle method was added (Di Stefano et al. 2010). The fraction < 63 µm was operationally defined as the sum of fine silt and clay, which is typically associated with the majority of the soil organic matter (Hemkemeyer et al. 2018) and considered to be to be important for soil stabilization through interactions between fine particles and organic carbon (Bird et al. 2002).

TOC and TN contents of the soil were measured using an elemental analyzer (vario MICRO cube, Elementar Analysensysteme GmbH). The detection limits were TOC (1 mg g^−1^) and TN (0.1 mg g^−1^) and it was not possible to determine TN content in samples younger than 54 years. Soil samples contained a significant amount of carbonates, which were removed prior to analysis via acid fumigation with HCl vapours (Harris et al. 2001). Analyses of TOC and δ^13^C_TOC_ contents of dried soil material were conducted with an NC Elemental analyzer (ThermoQuest, Bremen, Germany) connected to an isotope ratio mass spectrometer (IR-MS Delta X Plus, Finnigan, Bremen, Germany).

Carbon, nitrogen, and phosphorus in microbial biomass (C_mic_, N_mic_, and P_mic_) were estimated using the chloroform-fumigation extraction method (Brookes et al. 1982, 1985, Vance et al. 1987). The extraction of fumigated soil followed the protocol for available nutrients described above. Nutrient concentrations in microbial biomass were calculated as the difference between concentrations in fresh soil extract and fumigated soil extract. Results were corrected for incomplete extraction of all C_mic_ (Kec = 0.45), all N_mic_ (Ken = 0.54), and all P_mic_ (Kep = 0.4) (Brookes et al. 1982, 1985, Vance et al. 1987).

Tetraether lipids were extracted from six forefield soils (20 g wet weight, one pooled sample for each forefield site) after the protocol of Hopmans et al. (2004). The analysis was conducted solely as a complementary measure, without laboratory repetition. Therefore, caution was exercised when evaluating the data. Lipid extracts to assess the biomarkers were dried and stored frozen until analysis by liquid chromatography mass spectrometry (Becker et al. 2015). Tetraether lipids were quantified relative to a C46 tetraether injection standard and the relative response of a caldarchaeol standard. The compounds measured include isoprenoidal glycerol dibiphytanyl glycerol tetraethers having zero to three rings (iGDGT0-3; m/z 1302, 1300, and 1298), crenarchaeol (m/z 1292, biomarker for marine archea), and branched tetraethers produced by soil bacteria having varying degrees of rings and methylation (m/z 1018, 1020, 1022, 1032, 1034, 1036, 1046, 1048, and 1050). iGDGT having four rings (iGDGT4) was below the detection limit. The BIT index (branched and isoprenoid tetraethers index), which is a proxy for relative contribution of terrestrial and marine organic matter input to sediments, was calculated as a ratio of the sum of all measured branched tetraethers versus crenarcheol (Schouten et al. 2013).

### Soil enzymes analysis, DNA amplicon sequencing, and qPCR

Extracellular enzymatic activities were measured for five soil enzymes responsible for organic carbon, nitrogen, and phosphorus utilization with standard fluorometric technique (Marx et al. 2001) modified by Bárta et al. (2013). The activity of extracellular enzymes was determined in water extracts. Briefly, 0.5 g of soil was homogenized in 50 ml of distilled water via an ultrasonication. The soil suspensions (200 µl) were then transferred to a 96-well microplate. Then, 50 µl of substrates labeled with 4-methylumbelifferone (MUB) or 7-amino-4-methylcoumarin (AMC) were added. Standard curves were measured with MUB/AMC in soil slurries for every sample separately. Concentrations of MUB/AMC for standard curves were: 1, 5, and 10 µM. Microplates were incubated at room temperature for 2 h and fluorescence was measured every 30 min. All fluorescence measurements were performed on the microplate reader INFINITE F200 (Tecan, Germany) using the excitation wavelength of 365 nm and emission wavelength of 450 nm. Enzyme activities were expressed as nmol of liberated MUF/AMC per hour per gram of dry soil. The stoichiometric analysis of enzymes was done according to Hill et al. (2012) and is based on the assumption that microbes release extracellular enzymes in order to gain needed C, N, and P nutrients from complex organic substrates. The analysis compares ratios of C:N and N:P processing enzymes and the results may imply which nutrients are mainly limiting for the microbial growth and prosperity (Schmidt et al. 2016). Carbon processing enzymes (E_C_) comprise β-glucosidase (GLU) and cellobiohydrolase (CELL) activity, nitrogen processing enzymes (E_N_) are the sum of leucin aminopeptidase (ALA) and chitinase (CHIT) activity and phosphorus (E_P_) is represented by phosphatase (PHO) acitivity.

The amount of 0.25 g was used for DNA extraction using PowerSoil DNA isolation kit (MO BIO Laboratories, USA). The DNA was quantified fluorimetrically using Qubit (Thermofisher Scientific, UK) following the standard protocol and kit for dsDNA quantification. The aliquots of DNA extracts were sent to SEQme lab (Prague, Czech Republic) for the preparation of a library and sequencing using MiSeq platform. The Earth Microbiome Project (EMP) protocol was used for library preparation with modified universal primers 515FB/806RB (Caporaso et al. 2011) and ITS1F/ITS2 (White et al. 1990) for prokaryotic 16S rRNA and fungal ITS1 amplicons, respectively. The coverage of prokaryotic primer pair 515FB/806RB was additionally tested *in silico* using ARB Silva database release 128. The primer pair 515FB/806RB covers almost uniformly all major bacterial and archaeal phyla. Bacterial 16S rRNA raw pair-end reads (150 bp) were joined using ea-utils to obtain reads of ~250 bp length. Quality filtering of reads was applied as previously described (Caporaso et al. 2011). After quality filtering the sequences were trimmed to 250 bp. We obtained 720 625 bacterial and 1746 017 fungal sequences after quality trimming and filtering. Before picking the operational taxonomic units (OTU), the fungal ITS1 region was extracted from reads using ITSx algorithm (Bengtsson-Palme et al. 2013). Both 16S and ITS1 amplicons were trimmed to equal lengths in order to avoid spurious OTU clusters (Edgar 2013). Taxonomy was assigned to each read by accepting Silva119 taxonomy string of the best matching Silva119 sequence. Fungal reads were clustered to OTUs using open-reference OTU picking protocol (sequence similarity 98.5%) using UNITE ver. 8.0 database (Koljalg et al. 2013). Blast algorithm (e-value ≤ 0.001) was used for taxonomic assignment. FAPROTAX (Louca et al. 2016) and FUNguild (Nguyen et al. 2016) algorithm was then used for bacterial functional annotation and the fungal lifestyle assignments, respectively. Raw sequences were deposited at ENA under the project n. PRJEB51542.

Quantification of bacterial and fungal SSU rRNA genes was performed using the FastStart SybrGREEN Roche® Supermix and Step One system (Life Technologies, USA). Each reaction mixture (20 µl) contained 2 µl DNA template (∼ 1–2 ng DNA), 1 µl each primer (0.5 pmol µl^−1^ each, final concentration), 6 µl dH2O, 10 µl FastStart SybrGREEN Roche® Supermix (Roche, France), and 1 µl BSA (Fermentas, 20 mg µl^−1^). Initial denaturation (3 min, 95°C) was followed by 30 cycles of 30 s at 95°C, 30 s at 62°C, 15 s at 72°C, and completed by fluorescence data acquisition at 80°C used for target quantification. Product specificity was confirmed by melting point analysis (52–95°C with a plate read every 0.5°C) and amplicon size was verified with agarose gel electrophoresis. Bacterial standards consisted of a dilution series (ranging from 10^1^ to 10^9^ gene copies µl^−1^) of a known amount of purified plasmid where PCR product using the SSU gene-specific primers 341F/534R (Muyzer et al. 1993) was inserted. *R*^2^ values for the standard curves were > 0.99. Slope values were −> 3.37 giving an estimated amplification efficiency of > 93%. The qPCR conditions for fungal quantification were as follows: initial denaturation (10 min, 95°C) followed by 40 cycles of 1 min at 95°C, 1 min at 56°C, 1 min at 72°C, and completed by fluorescence data acquisition at 72°C used for target quantification. Fungal DNA standards consisted of a dilution series (ranging from 10^1^ to 10^7^ gene copies µl^−1^) of a known amount of purified plasmid where PCR product using the SSU gene-specific primers nu-SSU-0817–5′ and nu-SSU1196-3′ (Borneman and Hartin 2000) was inserted. *R*^2^ values for the fungal standard curves were > 0.99. The slope was between −3.34 and −3.53 giving estimated amplification efficiency between 95% and 93%, respectively. Detection limits for the assays (i.e. lowest standard concentration that is significantly different from the nontemplate controls) were less than 100 gene copies for each of the genes per assay. Samples, standards and nontemplate controls were run in duplicates. To deal with potential inhibition during PCR the enhancers BSA and DMSO were added to the PCR mixture. Also, several dilutions (10x, 20x, 50x, 100x, and 1000x) for each sample were tested to see the dilution effect on Ct values. Alpha diversity metrics the Chao1 richness (measurement of OTUs expected in samples given all the bacterial/fungal species that were identified in the samples), were calculated after rarefying all samples to the same sequencing depth of 3200 and 2800 sequences for bacterial and fungal community, respectively (Chao 1984).

### Statistical analyses

The effect of the northern versus southern forefield on soil parameters and on parameters changes in time were tested using a generalized linear mixed-effect model with Gamma distribution and logarithmic link function and a likelihood-ratio test, which was run in R version 4.0.2 (R Core Team 2020) in R package *stats*. The zone identity for each forefield was used as a random effect (i.e. eight groups). The significant differences in time were evaluated separately for each forefield using Tukey’s HSD *post hoc* tests in the R package *multcomp* to examine pairwise differences between the zones (different soil ages). Spearman correlation coefficients were determined in R package *ggpubr* to assess how strongly chosen parameters were related to each other. The effect of forefield on the total content of chosen soil elements determined by XRF and on labile compounds was visualized using principle component analyses in CANOCO for Windows 5.0 (Ter Braak and Šmilauer 2012) with zone (age) as a covariate. In all analyses from CANOCO, only the adjusted explained variation is presented in the text. In CANONO, variation partitioning was also applied to quantify the unique effects of TOC, bedrock (total content of elements in soil matrix determined by XRF—Si, Ca, Al, K, Fe, Mg, P, and S) and available nutrients (DOC, P-PO_4_^3−^, Nmin, Ca^2+^, Mg^2+^, and K^+^) on the microbial quantity (C_mic_, bacteria, and fungi) and quality (CNP processing enzymes), separately during 79 and 10 000 years of soil succession. Subsequently, a forward selection procedure was also performed to identify the single parameters that best explain the microbial quantity and quality and only *P*-values adjusted with Holms correction were considered. Both analyses were run with zone (age) as covariate. To estimate the beta-diversity in soil microbial communities and in their functional analyses, nonmetric multidimensional scaling (NMDS) ordinations were generated using CANOCO on the basis of Bray–Curtis dissimilarities of square-root transformed relative abundances of OTUs.

## Results

### Soil properties

**Figure 2.**
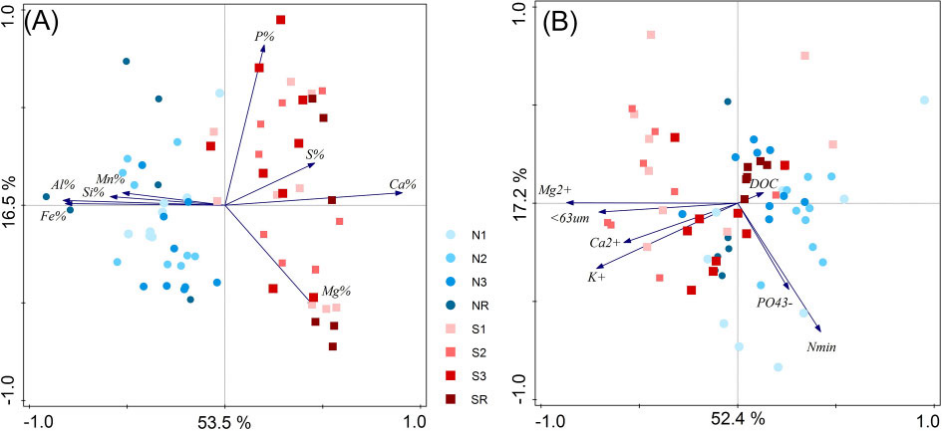
RDA ordinations of geochemical parameters of investigated soils constrained by the attribution to the forefield: **(A)** total content of main soil elements determined by XRF analysis (pseudo-F = 45.3, *P* < .01), **(B)** Water extractable nutrients and fine soil particles < 63 µm (pseudo-F = 15.7, *P* < .01). Soil age (Zone) was used as a covariate for both analyses.

XRF analysis of main soil elements has identified Si to be a prevailing element in the soil matrix of both forefields (from 13.6% to 22.5%), followed by calcium (from 3.5% to 8.3%) and/or aluminium (from 2.6% to 6.0%) (Table S1, Supporting Information). The significant difference in the content of most soil elements between northern and southern forefield corresponds to the overall lithological distinction described in detail within the methods (Fig. 2A; Table S1, Supporting Information). This geochemical distinction is further evident in the higher amount of water extractable cations and in higher content of fine soil particles < 63 μm of the more easily weatherable sedimentary rocks, which are present in the glacial till of the southern forefield (Fig. 2B; Table S2, Supporting Information).

**Figure 3.**
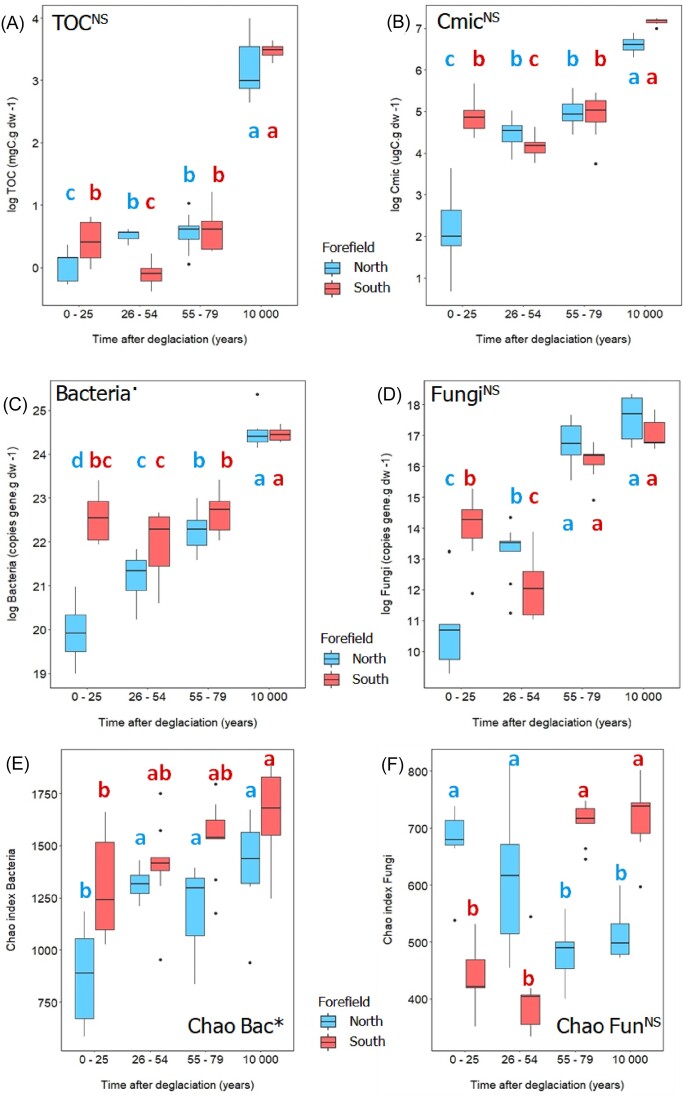
Effect of forefield and time on the microbial quantity determined by generalized linear mixed-effect model with Gamma distribution: **(A)** total organic matter, **(B)** carbon in microbial biomass, **(C)** amount of bacteria, **(D)** amount of fungi, **(E)** Chao index of bacteria, and **(F)** Chao index of fungi. Asterisks next to the title indicate significant difference between the forefields (* ∼ *P* = .01–.05, NS ∼ not significant). Different letters indicate significant differences among sites of different age within each forefield (*P* < .05, multiple comparisons using Tukey’s *post hoc* test). Boxplots visualize summary statistics (the median and the first and third quartiles) with whiskers and outlying points. Northern (red) and southern (blue) sites are described together with samples age: N1 and S1 = 0–25, N2 and S2 = 26–54, N3 and S3 = 55–79, and NR and SR = 10 000.

The higher proportion of fine particles < 63 µm in the soil matrix correlates with a higher soil moisture observed in the whole southern forefield (*r* = 0.7, *P* < .05; Table S2, Supporting Information). Besides the geochemical distinction, forefields did not significantly differ in pH, TOC, TN, or water extractable C and P, but they differed in water extractable N across all sites (Table S2, Supporting Information). All samples were alkaline with pH between 8.28 and 9.81. TOC levels showed very distinct trends in time within each forefield. TOC gradually increased with age on the northern forefield, from low values at the N1 site (0.11%) to high values at the NR site (2.78%) (Table S2, Supporting Information). In contrast, on the southern forefield, TOC was higher at the S1 site (0.16%) than at the N1 site (0.11%), decreased at the S2 site (0.09%), and grew in the later stages (S3 0.19%, SR 3.25%) (Fig. 3A; Table S2, Supporting Information). TN was below the detection limit in sites younger than 54 years and then increased on both forefields (Table S2, Supporting Information). All water extractable nutrients generally increased with time, only SO_4_^2−^ had the opposite trend. Both forefields contained a proportion of sulphate minerals, which released especially high amounts of SO_4_^2−^ on the S1 site together with water extractable Ca, Mg, and K (Table S2, Supporting Information).

The stable C isotope composition of TOC (δ^13^C_TOC_) varied from −20.8‰ to −24.3‰ (Table S3, Supporting Information). TOC samples collected on the southern forefield were typically more depleted in ^13^C than on the northern forefield, and N1 site exhibited the highest δ^13^C_TOC_ values (−20.8‰). The interpretation of the biomarker data is constrained by the limited replication, as only one pooled sample was used for each forefield site. However, despite this limitation, we have included the data in order to propose a broader range of explanations for the various successional developments observed. The BIT index on the northern forefield steadily increased with soil age (0.39–0.59; Table S3, Supporting Information), suggesting increasing inputs of terrestrial (brGDGT) relative to pelagic/glacial (crenarchaeol) OM over time (Hopmans et al. 2004, Brady and Daniel 2013). In contrast, the BIT index was consistently high on the southern forefield (> 0.69; Table S3, Supporting Information) suggesting relatively higher inputs of OM from terrestrial sources. The organic matter on the southern forefield generally comprised higher content of mono- and disaccharides, fatty acids, and sitosterol and brassicasterol (Table S3, Supporting Information).

### Microbial quantity and stoichiometry

Distinct temporal trends in TOC dynamics within each forefield were reflected in (i) microbial abundance expressed as C_mic_, (ii) the quantity of bacteria and fungi expressed as SSU gene copies per gram of dry soil, and (iii) bacteria species richness (Fig. 3). These microbial parameters differed the most between the youngest sites (i.e. N1 and S1). Values were the lowest at N1 sites, whereas southern S1 sites were characterized by significantly higher microbial biomass and bacterial and fungal abundance (Fig. 3). A significant drop of microbial biomass was observed at southern S2 sites, thus the drop in microbial quantity was significant only for fungi, not for bacteria (Fig. 3). The trends and values in the older sites (> 54 years) were similar in both forefields (Fig. 3B–D). Bacterial species richness was equivalent or increased with time on both forefields (Chao index: northern forefield from 890 to 1396, respectively, southern forefield from 1306 to 1648, respectively) (Fig. 3E), and was consistently higher in the southern forefield. However, fungal species richness in the youngest plots showed a completely different trend between the two forefields (Fig. 3F). In the southern foreland, the Chao index was significantly lower in the youngest plots S1 (679) and S2 (616) compared to the oldest plots S3 (480) and SR (513). The fungi in the northern foreland showed a completely opposite trend (Fig. 3F).

**Figure 4.**
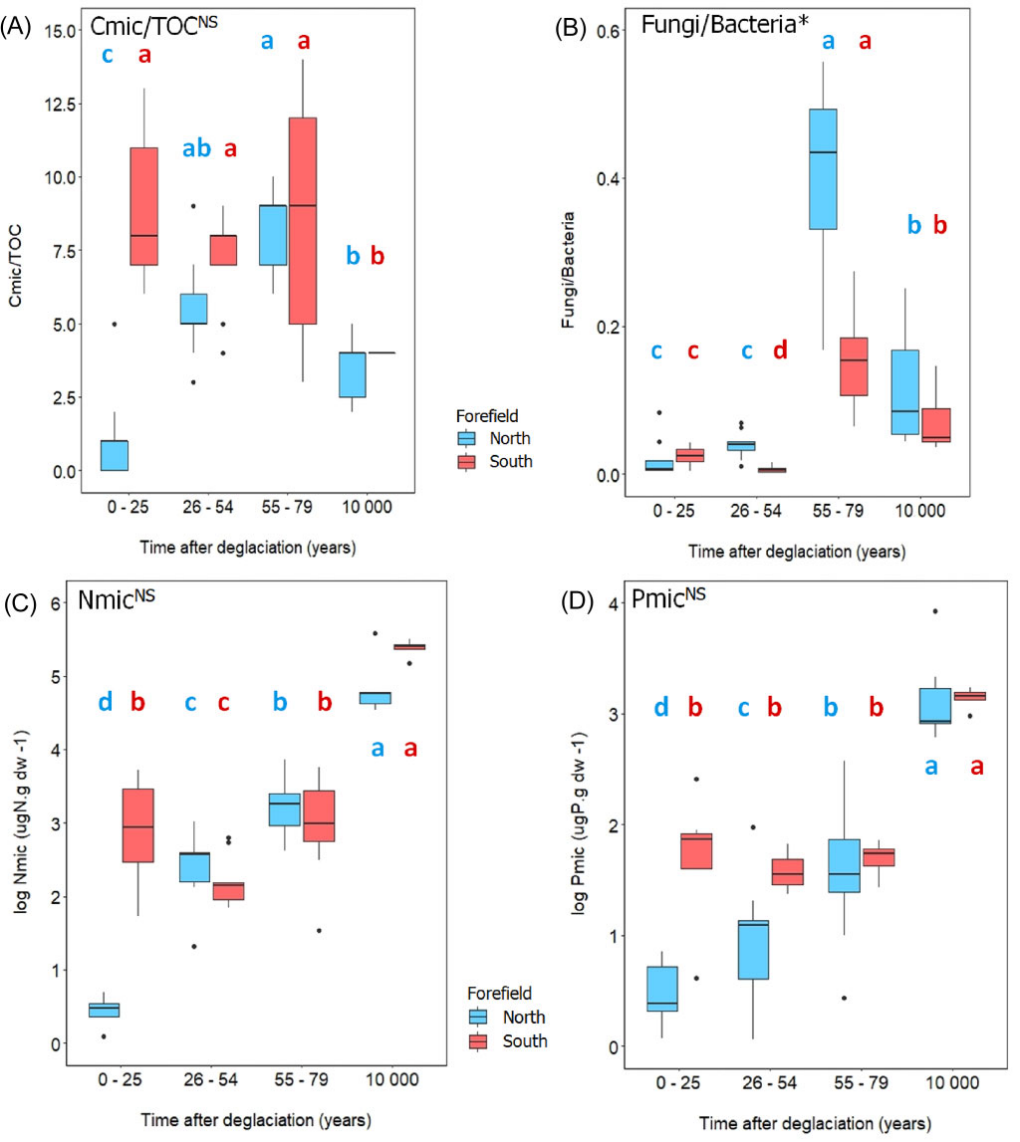
Effect of forefield and time on the microbial quantity determined by generalized linear mixed-effect model with Gamma distribution: **(A)** C biomass to TOC, **(B)** fungi to bacteria ratio, **(C)** nitrogen in microbial biomass, **(D)** phosphorus in microbial biomass. Asterisks next to the title indicate significant difference between the forefields (* ∼*P* = .01–.05, NS ∼ not significant). Different letters indicate significant differences among sites of different age within each forefield (*P* < .05, multiple comparisons using Tukey’s *post hoc* test). The boxplots visualize summary statistics (the median and the first and third quartiles) with whiskers and outlying points. Northern (red) and southern (blue) sites are described together with samples age: N1 and S1 = 0–25, N2 and S2 = 26–54, N3 and S3 = 55–79, and NR and SR = 10 000.

Forefields also differed in C_mic_ normalized to TOC, with extremely low values at the N1 sites (9.6 ± 11.0), compared to the significantly higher values at the S1 sites (143 ± 64.8) (Fig. 4A). However, similarly to TOC, most of all microbial parameters converged at N3 and S3 sites (after 79 years) and the forefields develop similarly in later stages. Fungi to bacteria ratio was very low in the youngest sites of both forefields (N1, N2, S1, and S2; from 7.24 × 10^−5^ to 4.00 × 10^−4^), indicating the general prevalence of bacterial over fungal communities. In later stages, especially in S3 sites (1.54 × 10^−3^), an expansion of fungi was observed (Figs 3D and 4B). Similarly to C_mic_, also N_mic_ and P_mic_ were considerably higher at the S1 site (21.7 µgN g dw^−^^1^ and 5.5 µgP g dw^−1^, respectively) compared to the N1 site (0.95 µgN g dw^−^^1^ and 1.5 µgP g dw^−1^, respectively) and did not change significantly at the S2 and S3 sites except of a decrease in N_mic_ at S2 sites, while they gradually increased on the northern forefield over time (Fig. 4C and D; Figure S1D, Supporting Information).

### Enzyme activity and stoichiometry

**Figure 5.**
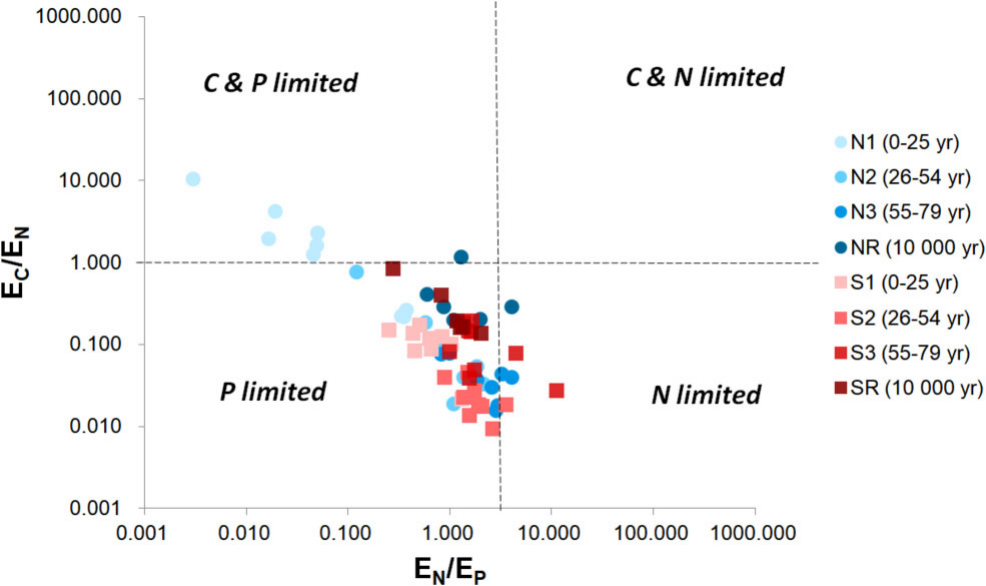
Stoichiometric analysis of C, N, and P processing enzymes (according to Hill et al. 2012). E_C_ = sum of carbon processing enzymes (β-glucosidase and cellobiohydrolase), E_N_ = sum of nitrogen processing enzymes (leucin aminopeptidase and chitinase), and E_P_ = phosphorus processing enzymes (phosphatase).

The sum of enzyme activity increased along the northern forefield, while there was no clear pattern on the southern forefield (Table 1). Despite remarkable differences in the sum of enzyme activity per gram of soil among N1 (31 nmol h^−1^ g^−1^) and S1 sites (359 nmol h^−1^ g^−1^), the values were similar when normalized to microbial biomass (2.82 nmol h^−1^ g^−1^ and 2.86 nmol h^−1^ g^−1^, respectively). At these youngest sites, phosphatase (PHO) was the main processing enzyme measured (84.3% and 59.5%, respectively), but its proportion decreased in time on behalf of N processing enzymes (Table 1). Carbon acquiring enzymes (GLU and CELL) had very low activities across both chronosequences until the reference sites. Stoichiometric analysis of CNP processing enzymes (Hill et al. 2012) revealed a clear distinction of the youngest deglaciated N1 and S1 sites in terms of potential nutrient limitation (Fig. 5). While the northern forefield appeared to be C and P colimited, the southern forefield was mainly P limited. Later, both forefields showed a similar trend of gradual shift from P limitation (S1 and N2 sites) to N limitation (S2, S3, and N3 sites) and finally to almost no nutrient limitation (S3, SR, and NR sites).

**Table 1.** Enzyme activities.

Site	Carbon enzymes	Phosphorus enzymes	Nitrogen enzymes	Sum of enzymes	Sum of enzymes/C_mic_
	(%)			(nmol h^−1^ g^−1^)	
**N1 (0–25 years)**	5.83 (0.68) b	84.3 (3.53) a	9.89 (3.42) b	31 (8.85) c	2.82 (0.70) a
**N2 (26–54 years)**	3.72 (0.67) bc	49.1 (5.12) b	47.2 (5.68) a	248 (36.1) b	2.95 (0.46) a
**N3 (55–79 years)**	2.69 (0.32) c	34 (4.04) c	63.3 (4.24) a	366 (19.5) b	2.66 (0.33) ab
**NR (10 000 years)**	17.7 (4.24) a	36.1 (5.40) bc	46.2 (4.95) a	876 (104) a	1.19 (0.16) b
**Forefield**	*	*	NS	NS	*
**S1 (0–25 years)**	3.83 (0.45) b	59.5 (3.44) a	36.7 (3.46) c	359 (45.9) b	2.86 (0.49) b
**S2 (26–54 years)**	1.42 (0.19) c	36.0 (2.76) b	62.5 (2.83) a	394 (39.5) b	6.03 (0.27) a
**S3 (55–79 years)**	6.04 (1.02) b	32.5 (3.83) b	61.5 (4.16) ab	375 (29.4) b	3.15 (0.67) b
**SR (10 000 years)**	10.9 (1.31) a	44.4 (4.47) ab	44.7 (5.58) bc	957 (106) a	0.75 (0.09) c

Statistics: effect of a forefield on enzyme activities (*** ∼ *P* < .001,** ∼ *P* < .01, * ∼ *P* = .01–.05, NS ∼ not significant) determined with a glmer model with a Gamma distribution and a logarithmic link function and a likelihood-ratio test. Effect of time on enzyme activities was evaluated separately for each forefield using Tukey’s HSD *post hoc* tests (different letters indicate significant differences; *P* < .05 between sites within the forefield). Brackets indicate ± standard error (within each site, *n* = 9), n.d. ∼ not detected.

Interpretation: carbon enzymes = sum of β-glucosidase (GLU) and cellobiohydrolase (CELL) activity, phosphorus enzymes = phosphatase (PHO) acitivity, and nitrogen enzymes = sum of leucin aminopeptidase (ALA) and chitinase (CHIT) activity.

### Effect of chemical parameters on microbial quantity and enzyme activity

**Figure 6.**
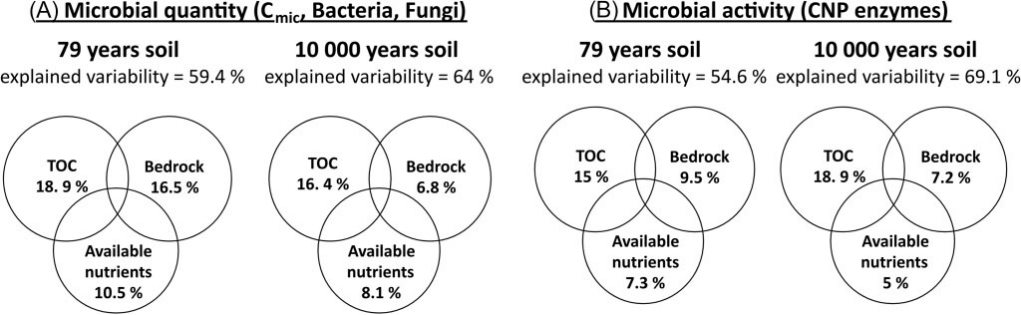
Variation partitioning analyses—the unique explained variability of TOC, bedrock, and available nutrients in **(A)** microbial quantity and **(B)** microbial activity. Analyses were made separately for 10 000 years and 79-years-old soil succession. TOC = total organic carbon, Bedrock = main soil elements measured with XRF (Al, Fe, Si, Mn, P, S, Mg, and Ca), and water extractable nutrients = DOC, PO_4_^3−^, Nmin, Ca^2+^, Mg^2+^, and K^+^. In all analyses age of the soil was used as covariate.

Both microbial quantity (microbial biomass, abundance of bacteria and fungi determined by qPCR) and enzyme activities were strongly positively correlated with soil age (C_mic_ r = 0.72, bacteria r = 0.68, fungi r = 0.82, and enzymes r = 0.66, all *P* < .001) and with TOC (C_mic_ r = 0.86, bacteria r = 0.75, fungi r = 0.81, and enzymes r = 0.63 respectively, *P* < .001). The exception was the soil development within 79 years in the S1–S3 sites, where the correlations of microbial parameters with age were not significant and the soil development was clearly driven by different parameters. Variation partitioning analyses of both forefields revealed that, together with time and TOC, bedrock type and available nutrients also played an important role. Specifically, bedrock (16.5%) and available nutrients (10.5%) explained together more variability than TOC (18.9%) during the first 79 years of soil succession (Fig. 6).

The forward selection of single environmental parameters revealed TOC to be the only explanatory variable of the microbial quantity (47.7%, pseudo-F = 58.3, *P* = .022) and microbial activity (53.6%, pseudo-F = 73.8, *P* = .022) across all sites. Interestingly, only microbial activity of the southern forefield was significantly influenced also by P-PO_4_^3−^ (7.4%, pseudo-F = 6.4, *P* = .03). This available phosphorus seemed to be especially important for the abundance of fungi, which showed a close correlation (r = 0.72, *P* < .001).

### Microbial community composition and functioning

After quality trimming and filtering, we obtained 737 010 (7046 OTUs) and 1521 308 (2774 OTUs) high quality sequences for bacterial and fungal communities, respectively. We were able to taxonomically affiliate 3932 bacterial OTUs (56%) and 602 fungal OTUs (22%) up to the genus level. In the fungal community, we found 535 OTUs (19%) that were taxonomically assigned only to the domain level. These fungal species might represent novel, unknown arctic species that are still not represented in recent fungal databases. Bacteria and fungi were rarefied to 3212 and 2866 sequences per sample, respectively.

**Figure 7.**
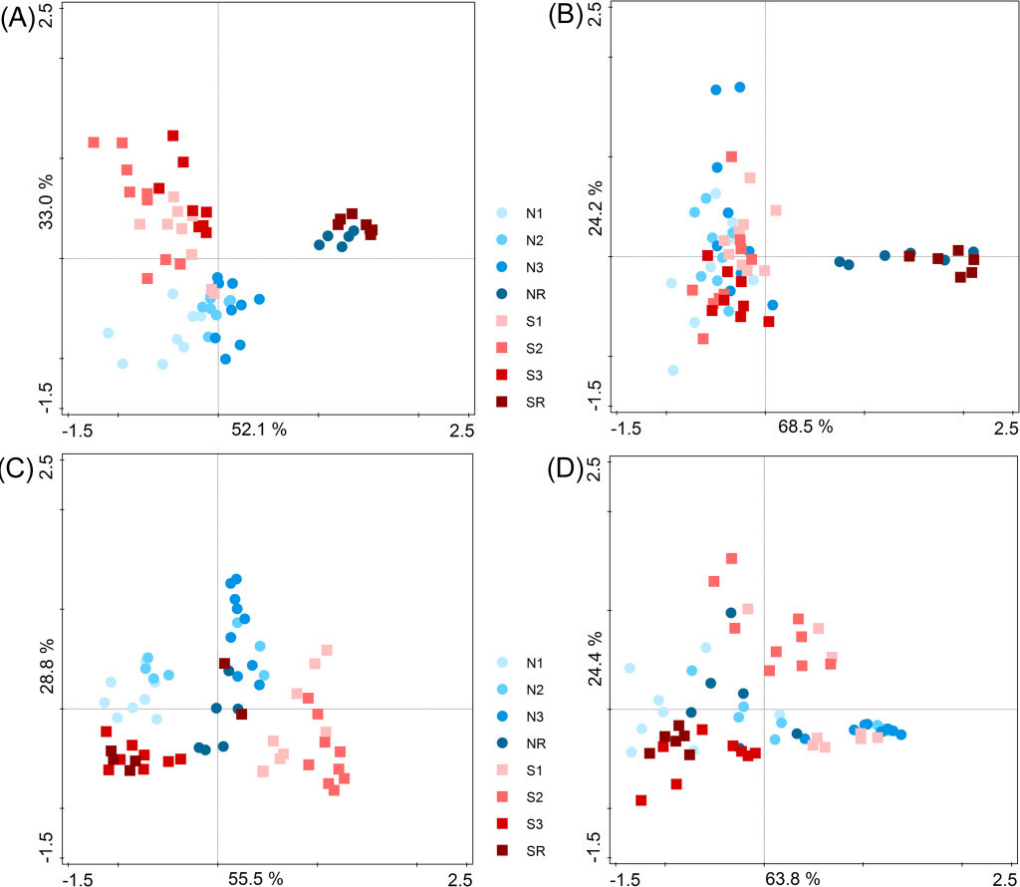
Bray–Curtis dissimilarities displayed with NMDS of **(A)** bacterial species (stress 0.059), **(B)** bacterial functional groups (stress 0.039), **(C)** fungal species (stress 0.128), and **(D)** fungal functional groups (stress 0.051). Samples age: 0–25 = N1 and S1 sites, 26–54 = N2 and S2 sites, 55–79 = N3 and S3 sites, and 10 000 = NR and SR sites.

NMDS analysis showed that the bacterial communities were different between the early and late stages (10 kyr) and were also different between the two forefields (Fig. 7A). In contrast, functional diversity was only different between the early stages and the late (10 kyr) stage but was not as markedly different between the forefields. Thus, our data showed that (i) although the bacterial communities of the early-stage forefields were distinct, they exhibited a higher degree of functional overlap and (ii) that the two forefields became more similar in both species composition and function after about 10 kyr. This trend of community convergence was consistent with the data from chemical analyses of the soils of the two forefields (Table S2, Supporting Information).

Functional analyses further showed critical differences in trends of specific fungal lifestyles, mainly the proportions of lichenized fungi between the northern and southern forefield (Figure S2, Supporting Information). Lichenized fungi in the youngest southern S1 sites represented 55% of the community, on average, and steadily decreased with time. In contrast, they represented only 12% in the youngest northern N1 sites and gradually increased from N1 to N3 sites.

The high proportion of OTU of lichenized fungi at the youngest southern S1 sites was accompanied by relatively higher proportions of cyanobacteria and diazotrophs (Figure S1, Supporting Information). These functional guilds decreased with time on the southern forefield while an opposite trend was seen on the northern forefield. This can relate to decreasing trends of C and N flux to the soil in the southern forefield, presumably via lower CO_2_ and N_2_ fixation performed by cyanobacteria and diazotrophs.

## Discussion

Global warming has accelerated the retreat of glaciers in polar ecosystems and the exposure of new terrain that may be subject to succession by many species, including microbial species. Studies in Alaska have shown that limited nutrient availability on poor geological substrates after the glacier retreat may be more important for the early microbial succession than climatic conditions, such as temperature and precipitation (Schmidt et al. 2016). These studies have also highlighted that the influence of local climate is difficult to separate from that of nutrient availability (Nemergut et al. 2005). Both questions remain unanswered because the available data were mostly compared between distant glacier forefields, where the influence of climatic conditions could not be appropriately filtered out (Lazzaro et al. 2009, Bajerski and Wagner 2013, Alfaro et al. 2017).

Our study provides, for the first time, a detailed insight into the biogeochemical and microbial succession in two closely spaced forefields of the retreating Nordenskiöldbreen (Svalbard, Norway) with similar climatic conditions but different bedrock geology (Fig. 2A). Given the close proximity of the two forefields, this study offers a unique possibility to test the hypothesis that the substrate quality is critically important for microbial succession during the first decades after deglaciation (Schmidt et al. 2016) and may considerably influence the development of microbial communities and biogeochemistry in later successional stages of soil evolution.

### Effect of mineral substrate on nutrient availability

Our study confirmed that different substrate parameters and nutrient availability resulted in different soil succession of the microbial community in nearby forefields. The most pronounced differences were in mineral substrate composition (Fig. 2). While the mineral substrate of the northern forefield was dominated by glacially formed rocks and thin glacial deposits with silicate rocks, the southern forefield was completely covered by thick glacial deposits with high proportion of carbonates. Carbonate sediments typically weather more rapidly and can increase the content of fine soil particles < 63 µm, leading to higher soil moisture (Table S2, Supporting Information) and higher nutrient availability for microbes (Fig. 2B). The higher content of simple saccharides and available fatty acids (Table S3, Supporting Information) and higher content of water-extractable Ca, Mg, and K (Fig. 2B; Table S2, Supporting Information) in the southern forefield indicated higher quality OM that can be more rapidly utilized by microorganisms, leading to higher microbial biomass in the southern forefield, especially at the youngest sites. Our data, therefore, show that substrate chemistry and nutrient availability are more important for microbial activity and early soil development (Figs 2 and 6) than the commonly reported TOC (Fig. 7A) (Tytgat et al. 2016, Kim et al. 2017).

However, the above-mentioned differences between the forefields do not provide us with any information on whether C, N, or P are available in sufficient quantities for microorganisms and whether they are limiting for their growth and development. To answer this question, we used an enzymatic model that provides information on the utilization of C, N, and P in the context of their limitation for microorganisms and their enzymatic activity (Fig. 5) (Hill et al. 2012). Enzymes of heterotrophic bacteria and fungi involved in C, N, and P processing showed distinct trends, especially in early successional stages on both forefields. We found a shift from P to N limitation in the southern forefield, whereas the northern forefield was initially more colimited by C–P and shifted to greater N limitation in later stages, which is consistent with a previous study (Bueno de Mesquita et al. 2020). Therefore, we hypothesize that the lower complexity and higher quality of C-rich organic material on the southern forefield required less investment by microorganisms in C enzymes to obtain energy and carbon, which allowed microbes to grow faster in the early stages but resulted in limitation of N and P. This can also be observed in different microbial biomass and abundance of bacteria and fungi in these forefields. The later shift to N limitation on both forefields corroborates other observations and a general paradigm of higher N requirements with more developed soils, among others, in association with increasing vegetation cover (Vitousek et al. 1993, Bueno de Mesquita et al. 2020). The different character of the organic material was further supported by an increasing BIT index and decreasing δ^13^C with age in the northern forefield, indicating gradual accumulation of soil-derived organic material, as could be expected along a classical chronosequence. These findings are consistent with reports of taxa mainly from supra- and subglacial habitats in front of the Damma Glacier, Switzerland (Rime et al. 2016a) or a gradual transition from glacial to edaphic taxa in front of the Fourcade Glacier, maritime Antarctica (Gyeong et al. 2021). We are aware that the inclusion of biomarker data with low replication (one pooled sample for each forefield site) needs to be justified. Based on our results, we would like to suggest that biomarker distributions may help to unravel microbial assembly dynamics that are still poorly understood and may not be fully captured by measuring soil physico-chemical parameters alone.

### Different succession of the microbiome in the northern and southern forefield

In Arctic extreme environments, the successional pattern deviates from the classical model of directional change and species replacement (Matthews 1978, Svoboda and Henry 1987). Under excessive climatic stress, competition is reduced and directional, nonsubstituting succession is more common, with native species remaining and new species being added during succession (Svoboda and Henry 1987, Jones and Henry 2003). Previous studies from Svalbard glacier forefields suggest that colonization of both plants and root-bound fungi follow this model of directional, nonsubstituting succession (Hodkinson et al. 2003, Davey et al. 2015).

Our data show that the bacterial and fungal microbiomes of the northern and southern forefields evolved differently in biomass, composition, and activity during the early stages of deglaciation. This different development lasted roughly until the first 79 years after deglaciation. Thereafter, convergence occurred and the differences between the forefields almost disappeared (Figs 3–5 and 7). Total microbial biomass was significantly higher on the youngest S1 sites than on the N1 sites (Fig. 3B). Compared to the northern, the southern microbiome experienced a significant decline in both abundance and diversity over a relatively short period of time, probably related to the decline of fungi (Fig. 3D) and probably also related to a significant rebuilding of the fungal microbiome (Fig. 3F; Figure S2, Supporting Information). The Chao index showed very low fungal richness of the youngest southern sites (Fig. 3F), indicating a dominance of few fungal species. Thus, the later increase in species richness may be a result of community rearrangement. Fungal communities developed differently from the beginning on both forefields, and differences were also reflected in the relative abundance of specific functional groups of fungi. Lichen-forming fungi are often found in early successional stages after glacial retreat (Syers and Iskandar 1973, Zumsteg et al. 2012, Garrido-Benavent et al. 2020), and they also strongly and relatively rapidly dominated the microbiome of the southern forefield, which correlates with higher abundance of bacterial phototrophs (Figure S1, Supporting Information) and with higher levels of sitosterol and brassicasterol (Table S3, Supporting Information), biomarkers indicating algal and/or plant phytomass (Volkman 2003, Rontani et al. 2012). However, the lichen-forming fungi abundance in the fungal community declined significantly over time on southern forefield and they were gradually replaced by saprotrophs and partially ericoid species in the oldest sites after deglaciation (Selosse et al. 2018). Thus, the fungal microbiome of the southern forefield experienced more pronounced fluctuations in both abundance and diversity, and a significant rebuilding of the fungal microbiome was observed, indicating a direct evolution with partial replacement of fungal species compared to the northern forefield with a rather nonsubstituting succession, more common for the High Arctic (Svoboda and Henry 1987, Jones and Henry 2003).

In contrast to the fungal microbiome, bacterial abundance and richness increased gradually with soil development on both forefields, consistent with other studies (Schütte et al. 2010, Jiang et al. 2018, Gyeong et al. 2021). However, we found specific differences in some major functional groups that might be important for initial succession in soil. The microbiome of the northern forefield had a higher proportion of bacteria degrading more complex and less available compounds in the early stages of degradation. This was mainly reflected by a higher proportion of cellulolytic and chitinolytic bacteria (Figure S1, Supporting Information) and probably related to lower nutrient availability and higher C mining (Fig. 5; Table S2, Supporting Information). In contrast, the young southern forefield contained a higher proportion of phototrophic and nitrogen-fixing bacteria (Figure S1, Supporting Information), also supported by lower δ^13^C_TOC_ values (Table S3, Supporting Information). These two key functional guilds in the southern forefield mediate the flux of atmospheric C and N during early soil succession to microbial community. When they have sufficient P for energy production (Darcy and Schmidt 2016, Darcy et al. 2018), they ‘pump’ available C and N into the system, allowing other heterotrophic microbes to develop (Chapin et al. 1994). Indeed, more P_mic_ was measured on the southern forefield (Fig. 4D), suggesting that higher availability of P promoted the development of heterotrophic bacteria and fungi and increased the overall microbial biomass in the early stages of deglaciation compared to the northern forefield. Our findings are consistent with other studies that have found that P is essential for accelerating phototrophic growth (Darcy and Schmidt 2016, Bueno de Mesquita et al. 2020) and for microbial succession in general (Knelman et al. 2014). Thus, the two forefields evolved quite differently in terms of the number, composition, and functional potential of the fungal and bacterial microbiome in the early stages up to 79 years after glacial retreat.

### Different trends in convergence and redundancy of bacteria and fungi over time

Similarly to other studies (Rime et al. 2016b, Alfaro et al. 2017, Jiang et al. 2018, Garrido-Benavent et al. 2020, Vimercati et al. 2022), the trajectories of bacteria and fungi showed different patterns during succession (Fig. 7). As discussed above, the bacterial and fungal microbiomes were clearly affected by the geochemistry and nutrient availability in the youngest sites (Fig. 7). Only bacteria showed a gradual convergence with time, whereas the lack of a clear pattern of fungal assembly is consistent with previous studies (Brown and Jumpponen 2014, Castle et al. 2016). Fungal succession is thought to be controlled by stochastic processes (Jiang et al. 2018, Gyeong et al. 2021, Lin et al. 2022, Vimercati et al. 2022), which may be caused by their different dispersal possibilities or their close relationships with vegetation (Schmidt et al. 2014), together with high geomorphological and geological heterogeneity of the glacial forefields (Ozerskaya et al. 2009, Wojcik et al. 2020, Gyeong et al. 2021). Compared to the microbial composition, functional groups of bacteria and fungi were shaped much less by the geochemistry and exhibited partial functional redundancy.

In summary, in the northern forefield, most geochemical and microbial parameters gradually increased with time and may be considered as the *classical chronosequence* reported by most previous studies (Crocker and Major 1955, Chapin et al. 1994, Bernasconi et al. 2011). In contrast, in the southern forefield, nutrients and microbial biomass were already high at the youngest sites, leading to significant later fluctuations in bacterial and fungal abundance and diversity that last for approximately the first 79 years after deglaciation.

We suggest that after deglaciation in the southern forefield, the early microbial communities thrived on the higher availability of nutrients, most likely provided by the mineral substrate and microbial guilds involved in CO_2_ and N_2_ fixation (mainly lichenized fungi and oxygenic phototrophs) and, therefore, evolved much faster. Subsequently, nutrient depletion occurred rapidly, driving the habitat to nutrient limitation, and the microbial biomass again declined and rearranged in composition and in functional potential (e.g. lichen-forming fungi were replaced by saprotrophic fungi) as partially observed in another study (Schmidt et al. 2016). Our findings support the hypothesis that different bedrock may accelerate early soil succession after deglaciation, providing limiting nutrients during microorganism assembly, thereby promoting more rapid soil stabilization and providing higher quality organic matter. Our study also showed distinct patterns of bacterial and fungal trajectories during succession. The chemical parameters and microbiomes of both forefields converged gradually with time, while the fungi did not show a clear pattern.

## Supplementary Material

fiad104_Supplemental_Files
