# Supplementary material for: Mineral substrate quality determines the initial soil microbial development in front of the Nordenskiöldbreen, Svalbard
Source: FEMS Microbiol Ecol. 2023 Sep 2;99(10):fiad104. doi: 10.1093/femsec/fiad104 (PMC10689212; doi:10.1093/femsec/fiad104)
Supplement: fiad104_Supplemental_Files [file fiad104_supplemental_files.zip › Tables_Supp_data _rev_230803.docx]

| **Table S1** Elemental composition of soil matrix measured by XRF analysis | | | | |  |  |  |  |
| --- | --- | --- | --- | --- | --- | --- | --- | --- |
| **Site** | **Si [%]** | **Ca [%]** | **Al [%]** | **K [%]** | **Fe [%]** | **Mg [%]** | **P [%]** | **S [%]** |
| **N1 (0-25 yr)** | 20.06 (0.53) b | 3.95 (0.74) | 5.47 (0.30) ab | 2.40 (0.13) | 2.70 (0.19) | n.d. | n.d. | 0.03 (0.05) |
| **N2 (26-54 yr)** | 22.52 (2.06) a | 3.52 (1.29) | 5.99 (0.55) a | 1.54 (1.10) | 2.67 (0.21) | n.d. | 0.01 (0.01) | n.d. |
| **N3 (55-79 yr)** | 21.54 (1.66) ab | 3.97 (0.99) | 5.80 (0.60) ab | 1.78 (1.07) | 2.55 (0.21) | n.d. | 0.01 (0.01) | n.d. |
| **NR (10 000 yr)** | 16.85 (1.03) c | 4.20 (1.48) | 5.32 (0.52) b | 1.94 (0.22) | 2.50 (0.26) | 0.17 (0.37) | 0.01 (0.02) | 0.03 (0.02) |
| **Forefield** | ** | *** | ** | NS | ** | NS | NS | ** |
| **S1 (0-25 yr)** | 17.90 (0.96) ab | 6.97 (0.89) b | 4.22 (0.37) a | 1.85 (0.19) a | 2.15 (0.18) a | 0.40 (0.57) | 0.01 (0.01) b | 0.28 (0.16) a |
| **S2 (26-54 yr)** | 17.85 (0.89) b | 7.08 (0.91) ab | 4.23 (0.47) a | 1.90 (0.20) a | 2.17 (0.21) a | 0.12 (0.35) | 0.02 (0.02) ab | 0.07 (0.10) b |
| **S3 (55-79 yr)** | 19.04 (1.09) a | 7.21 (0.78) ab | 4.35 (0.42) a | 2.04 (0.33) a | 2.20 (0.21) a | 0.12 (0.33) | 0.04 (0.02) a | 0.01 (0.02) b |
| **SR (10 000 yr)** | 13.59 (0.61) c | 8.28 (0.75) a | 2.57 (0.16) b | 0.88 (0.09) b | 1.52 (0.07) b | 0.70 (0.71) | 0.01 (0.01) b | 0.03 (0.02) b |

**Statistics:** Effect of a forefield on soil parameters (*** ~ p < 0.001,** ~ p < 0.01, * ~ p = 0.01 – 0.05, NS ~ not significant) determined with a glmer model with a Gamma distribution and a logarithmic link function and a likelihood-ratio test. Effect of time on soil parameters was evaluated separately for each forefield using Tukey’s HSD post hoc tests (different letters indicate significant differences; p < 0.05 between sites within the forefield). Brackets indicate ± standard error (within each site, n = 9), n.d. ~ not detected.

| **Table S2** Basic soil properties and water extractable nutrients | | | |  |  |  |
| --- | --- | --- | --- | --- | --- | --- |
| **Site** | **pH (H_2_O)** | **moisture** | **soil particles <63um** |  | **TOC** | **TN** |
|  |  | **[g cm^3^]** | **[%]** |  | **[%]** | |
| **N1 (0-25 yr)** | 8.89 (0.01) b | 4.08 (0.84) b | 17.5 (13.4) b |  | 0.11 (0.01) c | n.d.^1^ |
| **N2 (26-54 yr)** | 8.98 (0.04) ab | 4.64 (0.70) b | 4.99 (2.93) c |  | 0.17 (0.01) b | n.d.^1^ |
| **N3 (55-79 yr)** | 9.07 (0.04) a | 6.98 (1.08) b | 15.7 (6.50) b |  | 0.18 (0.02) b | 0.01 (0.00) |
| **NR (10 000 yr)** | 8.38 (0.04) c | 18.5 (2.51) a | 41.8 (16.4) a |  | 2.78 (0.60) a | 0.16 (0.02) |
| **Forefield** | NS | ** | * |  | NS | NS |
| **S1 (0-25 yr)** | 8.72 (0.04) b | 9.99 (0.96) b | 22.3 (10.0) b |  | 0.16 (0.02) b | n.d.^1^ |
| **S2 (26-54 yr)** | 9.23 (0.05) a | 11.07 (1.62) b | 35.0 (14.3) ab |  | 0.09 (0.01) c | n.d^.1^ |
| **S3 (55-79 yr)** | 9.39 (0.07) a | 9.92 (0.74) b | 44.6 (6.61) a |  | 0.19 (0.02) b | 0.01 (0.00) |
| **SR (10 000 yr)** | 8.70 (0.03) b | 25.42 (1.84) a | 53.9 (10.1) a |  | 3.25 (0.15) a | 0.17 (0.02) |

| **Site** | **DOC** | **P-PO_4_^3-^** | **Nmin** | **S-SO_4_^2-^** | **Ca^2+^** | **Mg^2+^** | **K^+^** |
| --- | --- | --- | --- | --- | --- | --- | --- |
|  | **[μg g-1 soil dry weight]** | | | | | | |
| **N1 (0-25 yr)** | 11.1 (2.03) | 0.24 (0.05) b | 2.20 (0.29) b | 278 (61.3) a | 398 (96.6) a | 54.4 (12.4) a | 40.8 (7.82) a |
| **N2 (26-54 yr)** | 12.6 (2.52) | 0.25 (0.02) ab | 2.43 (0.23) ab | 13.7 (2.34) c | 62.7 (11.4) b | 6.12 (1.19) c | 7.76 (1.38) b |
| **N3 (55-79 yr)** | 22.9 (4.28) | 0.44 (0.07) a | 2.19 (0.16) b | 49.7 (23.6) b | 86.3 (4.35) b | 13.9 (1.85) bc | 35.2 (11.8) a |
| **NR (10 000 yr)** | 27.1 (3.56) | 0.30 (0.03) ab | 3.70 (0.61) a | 48.3 (14.0) bc | 174 (14.3) ab | 34.7 (1.94) ab | 37.2 (5.74) a |
| **Forefield** | NS | NS | **.** | NS | * | * | NS |
| **S1 (0-25 yr)** | 11.5 (5.19) | 0.09 (0.01) c | 0.72 (0.16) b | 1035 (164) a | 1122 (168) a | 418 (88.9) a | 143 (29.1) a |
| **S2 (26-54 yr)** | 7.65 (1.49) | 0.09 (0.02) c | 1.7 (0.25) a | 557 (146) a | 503 (121) b | 436 (123) a | 135 (35.1) ab |
| **S3 (55-79 yr)** | 20.5 (4.28) | 0.89 (0.12) a | 2.12 (0.17) a | 143 (25.1) b | 107 (15.2) c | 36.3 (6.25) b | 58.7 (7.83) b |
| **SR (10 000 yr)** | 22.5 (4.48) | 0.29 (0.03) b | 2.81 (0.03) a | 14.4 (5.03) c | 157 (11.7) c | 22.9 (1.99) b | 8.62 (0.62) c |

**Statistics:** Effect of a forefield on soil parameters (*** ~ p < 0.001,** ~ p < 0.01, * ~ p = 0.01 – 0.05, NS ~ not significant) determined with a glmer model with a Gamma distribution and a logarithmic link function and a likelihood-ratio test. Effect of time on soil parameters was evaluated separately for each forefield using Tukey’s HSD post hoc tests (different letters indicate significant differences; p < 0.05 between sites within the forefield). Brackets indicate ± standard error (within each site, n = 9), n.d. ~ not detected, ^1^ TN detection limit 0.1 mg g^-1^.

| **Table S3** Bioindicator analyses and δ^13^C_TOC_ | | |  |  |  |  |  |
| --- | --- | --- | --- | --- | --- | --- | --- |
| **Site** | **δ^13^C_TOC_** | **BIT index^1^** | **sugars** | **fatty acids** |  | **sitosterol** | **brassicasterol** |
|  | **[‰]** |  | **[detector response]** | |  | **[µ g lipid gC_org_^-1^]** | |
| **N1 (0-25 yr)** | -20.80 | 0.39 | 212 | 2108 |  | 23.5 | 8.3 |
| **N2 (26-54 yr)** | -23.13 | 0.46 | 636 | 1300 |  | 29.4 | 6.6 |
| **N3 (55-79 yr)** | -22.94 | 0.59 | 1060 | 2478 |  | 4.8 | 3.4 |
| **NR (10 000 yr)** | n.a. | n.a. | n.a. | n.a. |  | n.a. | n.a. |
|  |  |  |  |  |  |  |  |
| **S1 (0-25 yr)** | -23.80 | 0.79 | 930 | 3268 |  | 78.8 | 20.3 |
| **S2 (26-54 yr)** | -23.40 | 0.82 | 3030 | 821 |  | 68.8 | 10.5 |
| **S3 (55-79 yr)** | -24.34 | 0.69 | 5207 | 5479 |  | 117.6 | 15.7 |
| **SR (10 000 yr)** | n.a. | n.a. | n.a. | n.a. |  | n.a. | ; |

**Interpretation:** BIT index (0-1) = ratio of a sum of branched tetraethers and crenarcheol: 0 = input of marine or glaciál production of Traumarcheota, 1 = input of terrestrial production of Euryarcheota, sugars = available mono- and di- saccharides, sitosterol = plant sterol from higher plants, brassicasterol = algae sterol
