# Supplementary material for: Mineral substrate quality determines the initial soil microbial development in front of the Nordenskiöldbreen, Svalbard
Source: FEMS Microbiol Ecol. 2023 Sep 2;99(10):fiad104. doi: 10.1093/femsec/fiad104 (PMC10689212; doi:10.1093/femsec/fiad104)
Supplement: fiad104_Supplemental_Files [file fiad104_supplemental_files.zip › Figures_Supp_data_rev.docx]

**Supplementary material and methods**

**Results**


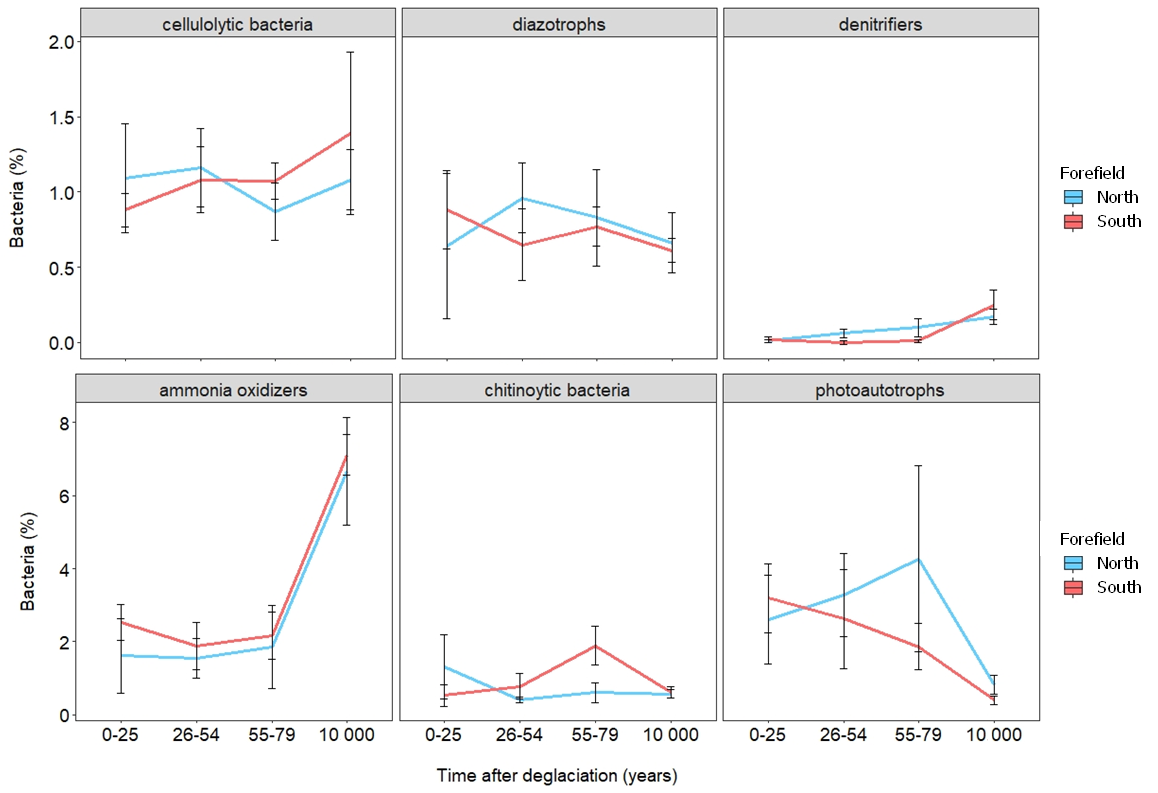


**Figure S1**: Succession of main bacterial functional groups in C cycle (cellulolytic bacteria, chitinolytic bacteria, photoautotrophs) and in N cycle (diazotrophs, denitrifiers, ammonia oxidizers). The functional annotation was based on FAPROTAX bioinformatic pipeline. N – north forefield, S – south forefield. Northern (red) and southern (blue) sites are described together with samples age: N1 and S1 = 0-25, N2 and S2 = 26-54, N3 and S3 = 55-79, NR and SR = 10 000.


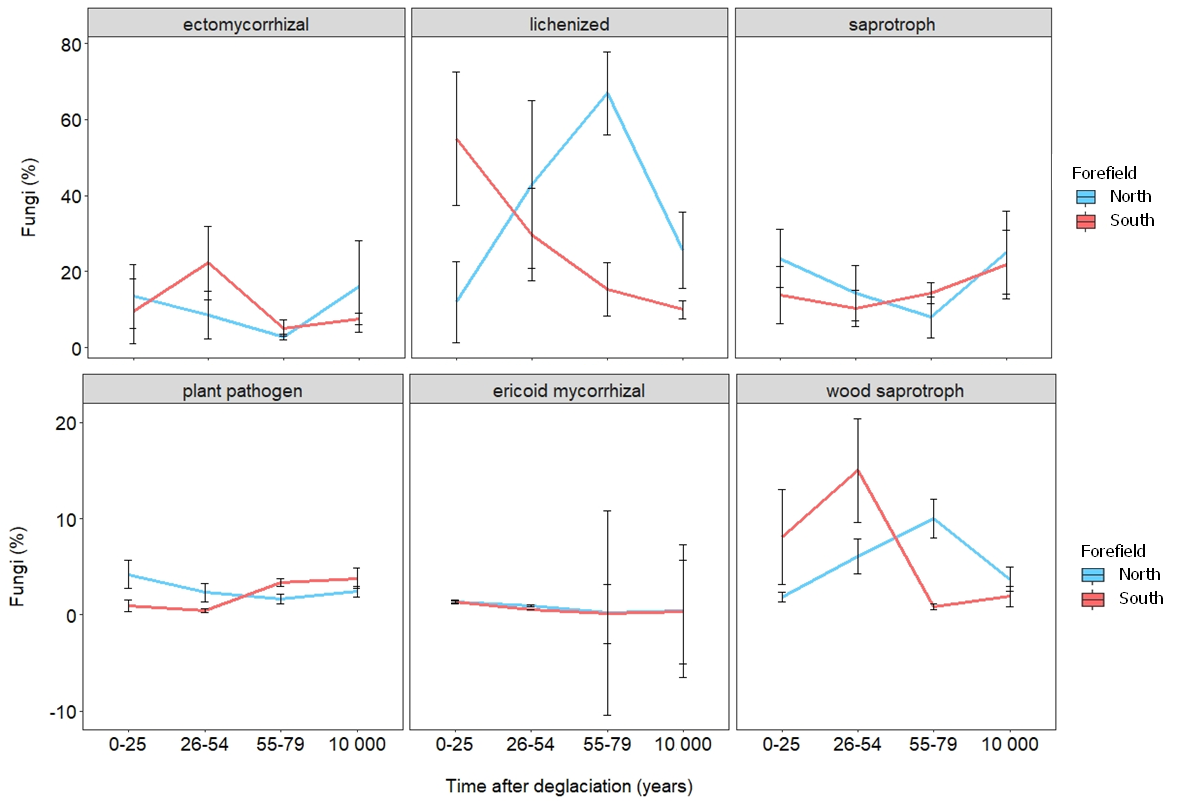


**Figure S2:** Succession of main fungal lifestyles based on FunGuild database. Northern (red) and southern (blue) sites are described together with samples age: N1 and S1 = 0-25, N2 and S2 = 26-54, N3 and S3 = 55-79, NR and SR = 10 000.
